# Standard English Dictionary

**Published:** 1891-10

**Authors:** 


STANDARD ENGLISH DICTIONARY. We have received some advance pages of this great work, which is to be issued by Funk & Wagnalls, 18 and 20 Astor Place, New York, and from what we can judge, at present, we should say that when finished it will be the completest and most reliable single volume dictionary in the language.

Among the eminent scholars who are in charge of the different departments of the work, may be mentioned, Professor Shaler of Harvard, Ex-Minister E. J. Phelps of Yale, Professor March of Lafayette College, President William R. Harper of the University of Chicago, Professor Simon Newcomb of Johns-Hopkins, Professor Max Muller, Professor Huxley, etc.

These editors are among the best known of American and English scholars ; each is an acknowledged authority in his particular sphere of learning. The Dictionary, from beginning to end, will be the work of scholars, and of experts in the different branches of the arts and sciences and in the different trades. Without reflecting unfavorably upon the work of others, we may be permitted to say that no dictionary of any language has had engaged upon it so many representative scholars.

Among the

DISTINGUISHING CHARACTERISTICS

of the Dictionary are the following ;

1. The etymology is placed *after* the definition.

2. In the definition of a word the *most common meaning* is given *first;* that is, preference is given to the "order of usage" over the historical order, usually followed in dictionary making. The aim is to remove everything that stands between the vocabulary word and the meaning that will be more generally sought after by the average reader.

3. The *scientific alphabet*, which has been prepared and recommended by the American Philological Association, and adopted by the American Spelling Reform Association, is *used in giving the pronunciation of words.* This department is under the direction of Professor Francis A. March, President of the American Spelling Reform Association, and who is recognized in Europe and America as one of the most eminent of living philologists.

4. *Disputed pronunciations and spellings* are referred, under the direction of Professor March, to a committee of fifty. This committee is composed of philologists in leading American, English, Canadian, Australian and East Indian Universities, and representative professional writers and speakers in English. By a simple system the form preferred by each committeeman will be indicated in the Preface to the Dictionary. The preference of this committee is advisory to Dr. March ; it is not mandatory.

5. If a vocabulary word is variously pronounced, we give first the pronunciation we prefer, then the pronunciation preferred by each of the other dictionaries.

6. A committee of five representative scholars will pass upon new words before they are admitted into the Dictionary.

7. The illustrative quotations are "*located*," that is, the volume and page where each is to be found are given.

8. Strictly obsolete and dialectic words, and such foreign words as are used only rarely in English literature, are placed in a Glossary in the Appendix, thus saving space in the Dictionary proper for tens of thousands of important living words that heretofore have been omitted from single volume dictionaries.

9. The different parts of each science are so treated, that the student can easily trace the definition of all its branehes, and have before him the full meaning of the science ; that is while the terms belonging to each branch or subordinate branch of a science are defined in their proper vocabulary places, the references to their superior and subordinate branches are so given that the definition of the science as a whole can easily be traced and collected, and when so collected will be found by the student to be a full and harmonious exposition of the entire science.

10. The church terms peculiar to the Roman Catholic Church, and to each of the Protestant denominations, and to other churches and religious organizations, are edited by a representative of the church or organization to which these words belong.

11. *Antonyms*, as well as synonyms, are given where this is thought important ; examples showing the proper use of *prepositions* are freely supplied in connection with different vocabulary words.

12. In the vocabulary, only proper names, or proper terms derived from them, are printed with initial capital letters, thus enabling any one to determine at a glance whether or not a word is to be written with an initial capital or small letter.

13. The work will contain *all* the words to be found in the latest Worcester, Webster, Stormouth and Johnson, and *nearly* 70,000 *more.*

The present seems to be a day of gigantic enterprises in dictionary-making, and we await with interest the appearance of this last.

